# Evaluation of comprehensive chromosome screening platforms for the detection of mosaic segmental aneuploidy

**DOI:** 10.1007/s10815-017-0924-4

**Published:** 2017-06-02

**Authors:** David Goodrich, Tongji Xing, Xin Tao, Agnieszka Lonczak, Yiping Zhan, Jessica Landis, Rebekah Zimmerman, Richard T. Scott, Nathan R. Treff

**Affiliations:** 1 Reproductive Medicine Associates of New Jersey, 140 Allen Rd, Basking Ridge, NJ 07920 USA; 2 Foundation for Embryonic Competence, 140 Allen Rd, Suite 300, Basking Ridge, NJ 07920 USA

**Keywords:** Segmental aneuploidy, Comprehensive chromosome screening, Next generation sequencing, Mosaicism, Preimplantation genetic screening

## Abstract

**Purpose:**

A subset of preimplantation embryos identified as euploid may in fact possess both whole and sub-chromosomal mosaicism, raising concerns regarding the predictive value of current comprehensive chromosome screening (CCS) methods utilizing a single biopsy. Current CCS methods may be capable of detecting sub-chromosomal mosaicism in a trophectoderm biopsy by examining intermediate levels of segmental aneuploidy within a biopsy. This study evaluates the sensitivity and specificity of segmental aneuploidy detection by three commercially available CCS platforms utilizing a cell line mixture model of segmental mosaicism in a six-cell trophectoderm biopsy.

**Methods:**

Two cell lines with known karyotypes were obtained and mixed together at specific ratios of six total cells (0:6, 1:5, 2:4, 3:3, 4:2, 5:1, and 6:0). A female cell line containing a 16.2 Mb deletion on chromosome 5 and a male cell line containing a 25.5 Mb deletion on chromosome 4 were used to create mixtures at each level. Six replicates of each mixture were prepared, randomized, and blinded for analysis by one of the three CCS platforms (SNP-array, VeriSeq NGS, or NexCCS). Sensitivity and specificity of segmental aneuploidy at each level of mosaicism was determined and compared between each platform. Additionally, an alternative VeriSeq NGS analysis method utilizing previously published criteria was evaluated.

**Results:**

Examination of the default settings of each platform revealed that the sensitivity was significantly different between NexCCS and SNP up to 50% mosaicism, custom VeriSeq, and SNP-array up to 66% mosaicism, and between NexCCS and custom VeriSeq up to 50% mosaicism. However, no statistical difference was observed in mixtures with >50% mosaicism with any platform. No comparison was made between default VeriSeq, as it does not report segmental imbalances. Furthermore, while the use of previously published criteria for VeriSeq NGS significantly increased sensitivity at low levels of mosaicism, a significant decrease in specificity was observed (66% false positive prediction of segmental aneuploidy).

**Conclusion:**

These results demonstrate the potential of NGS-based detection methods to detect segmental mosaicism within a biopsy. However, these data also demonstrate that a balance between sensitivity and specificity should be more carefully considered. These results emphasize the importance of vigorous preclinical evaluation of new testing criteria prior to clinical implementation providing a point of departure for further algorithm development and improved detection of mosaicism within preimplantation embryos.

## Introduction

Since the improvement in amplification strategies and the development of the ability to accurately screen for and diagnose aneuploidy in all 24 human chromosomes, contemporary comprehensive chromosomal screening (CCS) methods have become well developed and are now a common, routine part of infertility care. Given that approximately half of human preimplantation embryos are abnormal and the fact that aneuploidy rates increase drastically with advanced maternal age [1], these important advancements in PGS have allowed for improved outcomes in select patients undergoing in vitro fertilization (IVF) [2–5].

In particular, much attention has been given to the detection and screening of whole chromosome aneuploidy. A variety of methods including qPCR, array-CGH, and next generation sequencing (NGS) have been developed in order to accurately screen embryos for use in IVF. Namely, the development of high-throughput, massively parallel sequencing for use with CCS has been on the forefront of much research and is now common in many clinics. This advance in technology correlates to high accuracy screening of multiple samples while maintaining low costs.

With such advances in technology, it is now even possible to screen embryos for sub-chromosomal imbalances including inherited unbalanced translocations and segmental aneuploidies utilizing methods such as SNP-array and NGS [6–9] and the detection of small segments has been reported [10–13]. Recently, it has been reported that the frequency of clinically significant de novo segmental imbalances is higher than originally thought (2.5%) [14]. Recent data also suggests that the majority of segmental errors arise during mitosis, which leads to mosaicism [15]. Furthermore, recent data published by Kort et al. reported findings of several reciprocal segmental aneuploidies in discarded embryos that had been biopsied multiple times [16], leading us to suspect that segmental aneuploidy, when present, may be commonly found in a mosaic state.

The ability to detect segmental aneuploidy within a mosaic embryo may be possible when performing testing on a trophectoderm biopsy which contains multiple cells from the blastocyst. Mosaicism within the biopsy itself may result in altered, non-integer copy numbers and could be detectable with new CCS methodologies. This study sought to model the various levels of segmental mosaicism that might be observed in a typical trophectoderm biopsy and compare three current technologies with ability to detect sub-chromosomal imbalances to detect segmental aneuploidy in a mosaic sample.

## Materials and methods

**Fig. 1:**
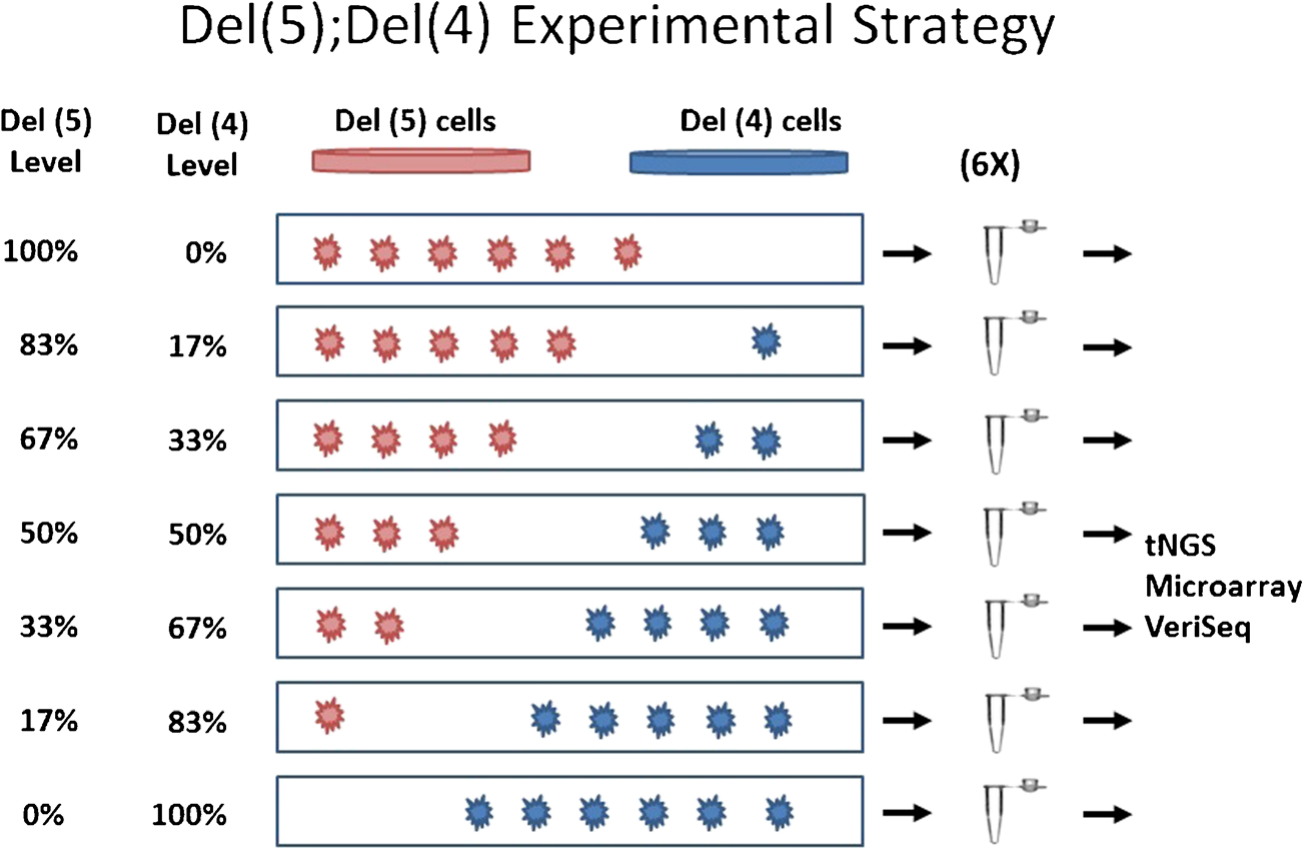
Mixture model experimental strategy illustration for preparation of samples involving a male del(4) cell line and a female del(5) cell line where inverse changes in levels of each aneuploidy are expected. Cells are mixed in a single tube in known ratios of six total cells (0:6, 1:5, 2:4, 3:3, 4:2, 5:1, and 6:0) to mimic various levels of mosaicism in a trophectoderm biopsy

In order to create positive controls for specific levels of mosaicism, two cell lines containing known segmental deletions, GM14131 (46,XX,del(5)(p15.1).ish del(5)(p15.33p15.1)(D5S23-).arr 5p15.33p15.1(68519-16362247)×1) and GM22601 (46,XY,del(4)(p15.2).arr 4p16.3p15.2(55665-25591051)×1), were purchased from Coriell Cell Repository (Camden, NJ). Each cell line was previously characterized for karyotypes by the supplier. The cells were then cultured and passaged once as recommended by the supplier. Individual cells were collected under a dissecting microscope and mixed together at specific ratios of six total cells (0:6, 1:5, 2:4, 3:3, 4:2, 5:1, and 6:0). Twelve replicates of each mixture were made and then equally and randomly divided between three CCS platforms for aneuploidy screening (Fig. 1).

Three CCS screening methods were examined in this study: (i) VeriSeq PGS (Illumina Inc., Santa Clara, CA), a commercially available method involving whole-genome amplification (WGA) and next generation sequencing (NGS) on a MiSeq; (ii) targeted next generation sequencing (NexCCS) (Foundation for Embryonic Competence Inc.), another commercially available method involving targeted amplification and next generation sequencing on an Ion Torrent Proton sequencer; and (iii) SNP-array, a whole-genome amplification-based method that uses arrays to assess aneuploidy [17]). Additionally, the first platform (VeriSeq NGS) was further evaluated utilizing previously published criteria by Vera-Rodriguez et al. [13]. Blinded computational segmental aneuploidy predictions were then made utilizing one of four criteria: (i) utilizing an in-house custom python algorithm to analyze the results from the Affymetix software (SNP-array); (ii) as recommended by the supplier utilizing the automatic aneuploidy calls made by Bluefuse Multi software (BlueFuse, Illumina Inc., version 4.2(20289)), termed “Default VeriSeq”; (iii) customized criteria for VeriSeq PGS (which examines every 10 Mb of amplicons, observes changes in the median copy numbers, and overrides automated calls made by Bluefuse Multi software), termed “custom VeriSeq”and as previously published [18]; and (iv) customized criteria for NexCCS, termed “NexCCS.”

After the predictions of segmental aneuploidy were made, samples were then unblinded and evaluated for consistency with expected results. Sensitivity was defined as the percentage of samples which were predicted as abnormal for the correct chromosome segment depending on which mixture was tested (i.e., del(4) or del(5)) and was determined for each chromosome (*n* = 12) at each of the respective mixture levels for each platform and its analysis settings. Specificity was defined as the percentage of samples where euploidy was predicted for all the chromosomes and segments expected to be normal or disomic (*n* = 42 for each method: the no. of true positives for each mixture level multiplied by the no. of sets of samples (2)). The performance of each platform was then compared using a chi-squared test for statistical significance at each mixture level for its sensitivity and overall specificity.

## Results

**Fig. 2:**
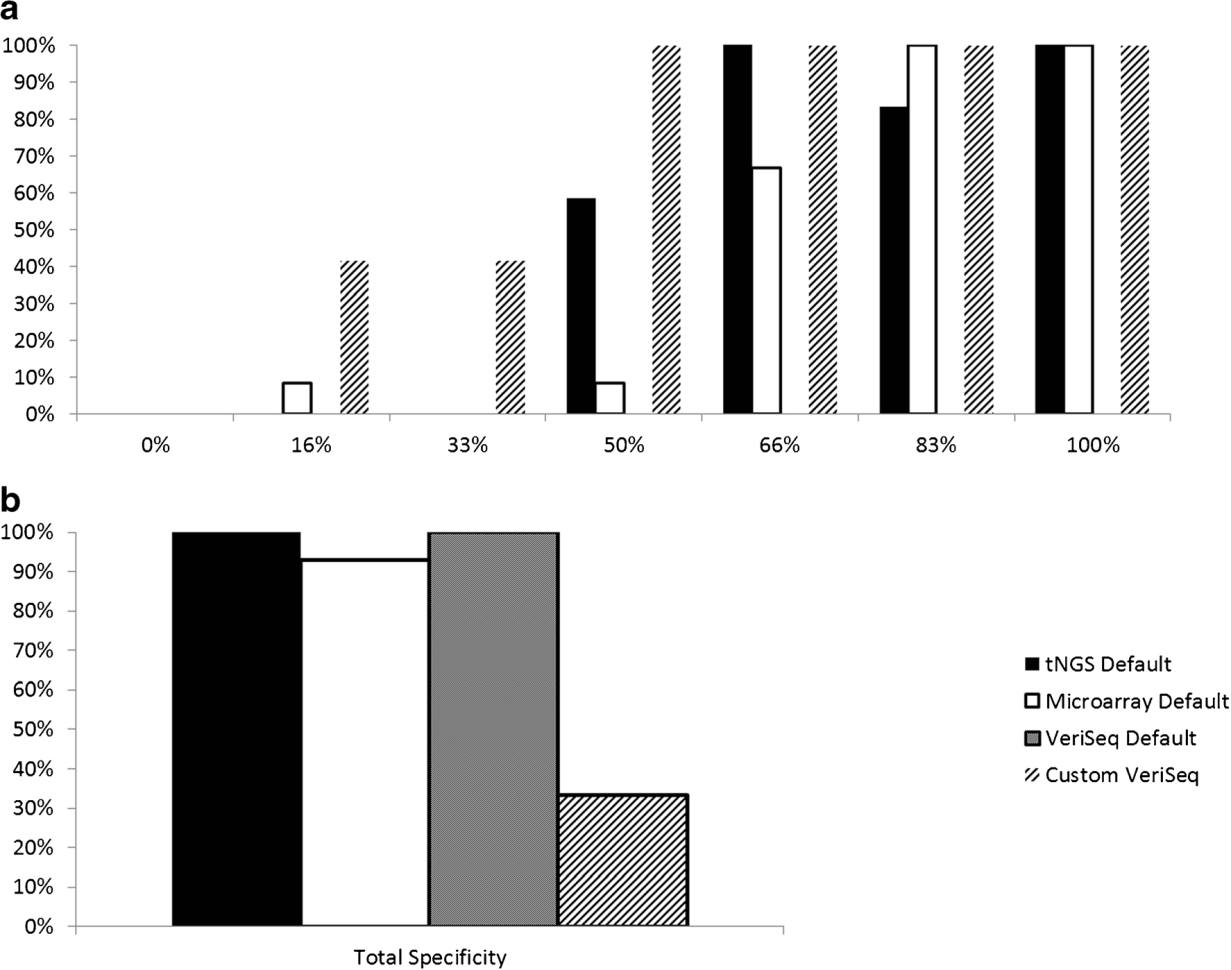
**a** Sensitivity across three sets of analyses for each mixture level: custom NexCCS, custom SNP-array, VeriSeq default settings, and VeriSeq with criteria defined by Vera-Rodriguez et al. [13] (custom VeriSeq). Sensitivity is based on detecting segmental deletions at each level. **b** Specificity across all samples for the same four analysis methods based on the frequency of detecting a normal copy number for each of the remaining chromosomes known to be uniformly normal

Analysis of the three CCS platforms demonstrated the ability to reliably predict an abnormality correctly at as low as 17% with custom VeriSeq, 50% with custom NexCCS, and 50% with SNP-array (Fig. 2). In all sample sets, increased detection of segmental errors was observed as the percentage of aneuploid cells increased in the mixture. Default VeriSeq settings, which utilize automated aneuploidy calls from Bluefuse Multi software do not report segmental aneuploidies and therefore did not detect segmental imbalances at any mixture level. Comparison of custom VeriSeq and NexCCS yielded significant differences at 17% (*p* = 0.0119), 33% (*p* = 0.0119), and 50% (*p* = 0.0119); however, there was no significant difference at 66% (*p* = 1), 83% (*p* = 0.139), or 100% (*p* = 1). Similarly, comparison between NexCCS and SNP-array yielded no significant statistical difference at 17% (*p* = 0.307) or at 33% (*p* = 1). Significant statistical differences were observed at 50 and 66% (*p* = 0.0093, *p* = 0.0284); however, as aneuploidy increased in the sample (83, 100%), no significant differences were observed (*p* = 0.139, *p* = 1). Lastly, comparison between SNP-array and custom VeriSeq resulted in statistical differences at 33% (*p* = 0.0119), 50% (*P* < 0.0001), and 66% (*p* = 0.0284), but no difference was observed at 88 or 100% (*p* = 1) (Fig. 2). The overall specificity was 93% for custom SNP-array (*p* = 0.0126), and 100% for custom NexCCS and Default VeriSeq methods of analysis (Fig. 2).

In contrast, when custom VeriSeq analysis criteria, as defined by Vera-Rodriguez et al. [13], were applied to the data set, significantly improved sensitivity of detecting aneuploidy was observed from 17 to 66% mosaicism levels (*p* < 0.05). Nevertheless, the subsequent improvement in sensitivity resulted in a greatly significant increase (*p* < 0.0001) in the rate of false positive segmental calls. The false positive rate increased from 7% (6/84), using SNP-array, 0% (0/84) with custom NexCCS and default VeriSeq analysis methods, to a stark 67% (56/84) when utilizing the custom VeriSeq criteria (Fig. 2). It is important to note here that WGA-based amplification methods such as VeriSeq may provide better coverage of the genome; however, this comes at a significant cost to specificity. Furthermore, while target-based methods may provide less coverage of the genome and thus decreased sensitivity, they will display better overall specificity. This limitation can also make the diagnosis of de novo segmental imbalances in regions not covered by the sequencing panel difficult. Further refinement of the panel or inclusions of more targeted regions may help in addressing this problem.

These results clearly demonstrate the importance of balance between sensitivity and specificity when considering criteria for detection of segmental aneuploidy in a mosaic sample. Overall, two thirds of the samples gave similar false positives when applying previously published custom analysis criteria, illustrating further need to carefully evaluate criteria prior to its implementation in a clinical setting.

**Fig. 3:**
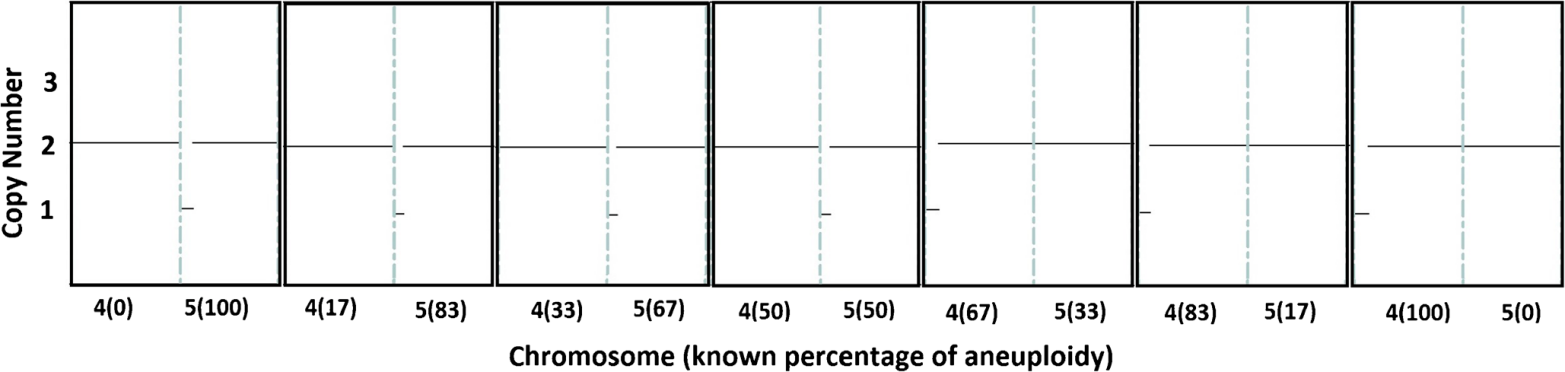
Example plots from SNP-array analyses of the segmental del(4) and del(5) six-cell mixture sets. As the level of aneuploidy increases in the sample, there is a concomitant change in the copy number values of the chromosome segments of interest

**Fig. 4:**
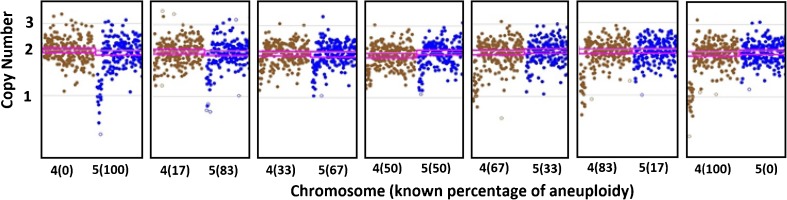
Example plots from NexCCS analyses of the segmental del(4) and del(5) five six-cell mixture sets. As the level of aneuploidy increases in the sample, there is a concomitant change in the copy number values of the chromosome segments of interest. This can be seen through the gradual migration of amplicons (represented by *blue* and *orange dots*) on chromosomes 4 and 5 in an inverse fashion

**Fig. 5:**
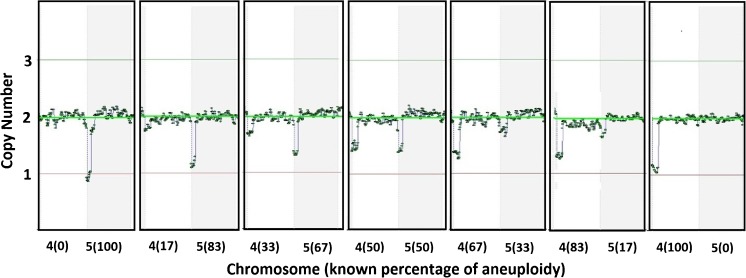
Example plots from VeriSeq NGS analyses of the segmental del(4) and del(5) six-cell mixture sets. As the level of aneuploidy increases in the sample, there is a concomitant change in the copy number values of the chromosome segments of interest

**Fig. 6:**
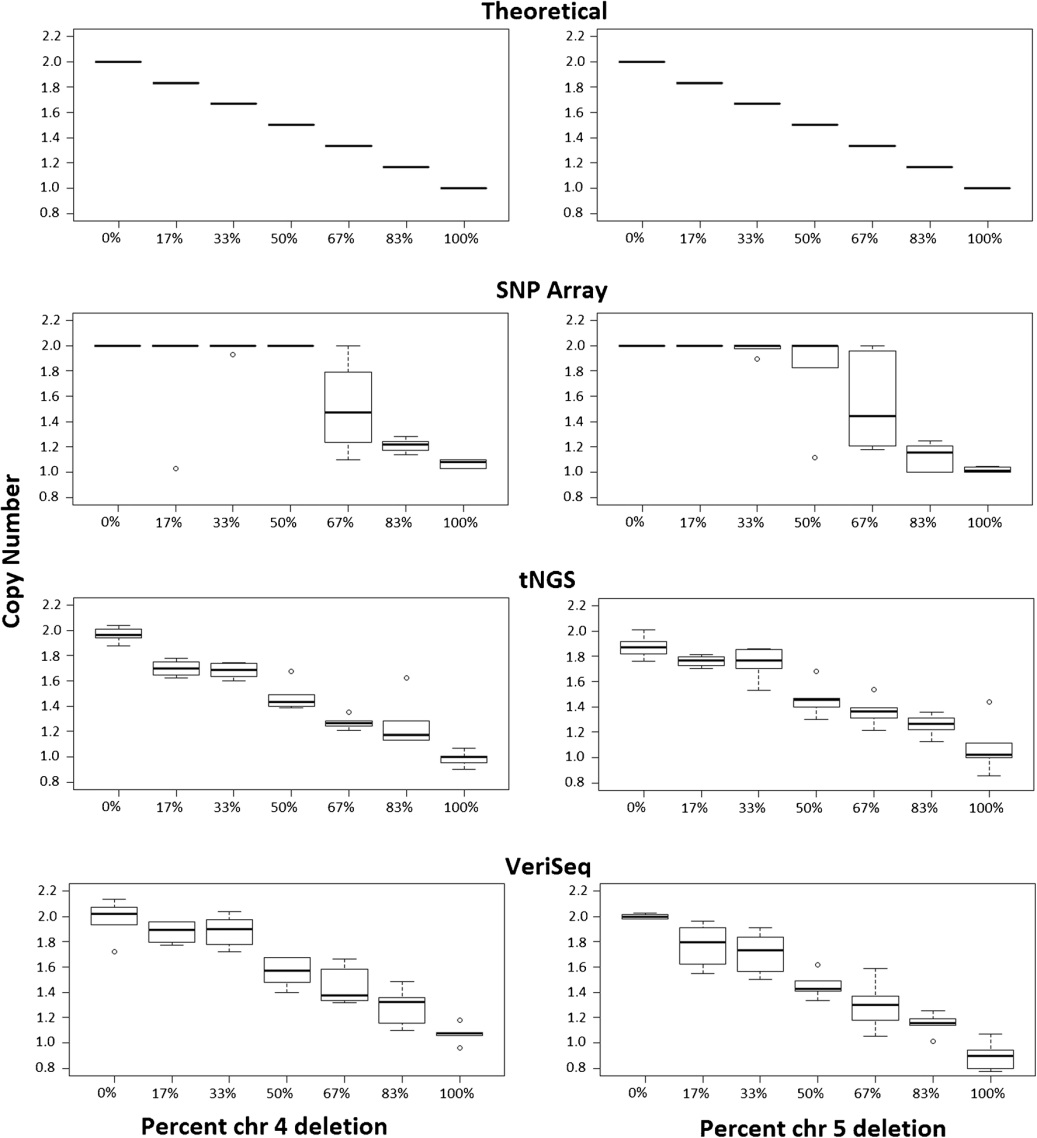
*Box* and *whisker plots* showing the distribution and variance of copy number assignments for target mosaic chromosome segments as the percent of spike-in aneuploidy increases in the sample with each respective platform (SNP-array, NexCCS, and VeriSeq NGS). As the level of aneuploidy increases in the sample, there is an overall decrease in the copy number of the chromosome segments of interest (4 and 5). Included is a theoretical box plot illustrating the expected copy number change based on the contribution of chromosomes from each sample

In order to illustrate the performance of each platform, example copy number plots for SNP-array, NexCCS, and VeriSeq are shown in Figs. 3, 4, and 5, which show the expected gradual change as the level of segmental aneuploidy increases in each mixture. SNP-array, which does not show intermediate copy numbers, only shows a change in the whole copy number assignments and migrates from disomy to monosomy. Reproducibility was considered by evaluating the distributions of copy number assignments for all replicates for both platforms and is shown in Fig. 6.

## Conclusions

Recently, research into the ability of contemporary CCS platforms to detect mosaicism has been given considerable attention. Embryonic mosaicism (both whole chromosome and segmental) is a complicated problem, and there are still many important factors that must be considered prior to implementation of screening in the clinic [19, 20]. Segmental aneuploidy in particular represents a unique challenge in that the detection limits are much lower than those observed with whole chromosome mosaicism [21]. Additionally, other factors such as the size and location of the duplication/deletion may also affect the predictive value of the individual biopsy as well as the clinical outcome of the embryo. Furthermore, the distribution of mosaicism within the embryo may also impact the accuracy of such a test, as by definition a sampling error of the embryo will exist when only single biopsy is taken.

However, while these issues may be relevant, the purpose of this study was to focus on the limits of detection of the three platforms; namely, the percentage of cells within a multi-cell sample that need to be aneuploid to allow detection, how often a platform can detect the abnormal cells, and how often artifacts of the technology result in incorrectly predicted abnormalities. This study also only focuses on segmental aneuploidies that are >5 Mb as this detection limit has been previously validated with NGS [6]. This experimental design strategy was the foundation of two previous studies, the first of which examined the ability of NGS and qPCR to detect whole chromosome aneuploidy in a mosaic sample [21], and the second which sought to develop an accurate method of qPCR-based CCS for uniform aneuploidy [20]. Although criteria for detecting mosaicism was recently described [13] and represents an important first step into the investigation of mosaicism, further refinement of criteria is needed. This is clearly illustrated by the fact that although a significant increase in the sensitivity of detection was seen, it resulted in a drastic loss (66%) of specificity. In that, in two thirds of the samples analyzed, a false positive was reported.

While defining the specificity and sensitivity of a method is important to the development of an accurate model of mosaicism, additional considerations must be given prior to implementation in the clinic as a diagnostic tool [22]. These include the distribution of mosaicism in the remaining embryo, the size of the segmental imbalance, the identity of the chromosome affected, and the actual clinical outcomes. Further preclinical testing should include evaluating multiple biopsies of the same embryo in order to establish the predictive value of a single biopsy for the remaining embryo [23]. Next, a prospective, blinded, non-selection study should be performed to establish positive and negative predictive values of a diagnosis for actual clinical outcomes [24]. Finally, new clinical interventions should work towards randomized clinical trials ultimately to establish the efficacy of a diagnosis of mosaicism as a predictor of reproductive outcome [15].
